# Thickness of epicardial and pericoronary adipose tissue measured using 128-slice MSCT as predictors for risk of significant coronary artery diseases

**DOI:** 10.1007/s11845-020-02339-8

**Published:** 2020-08-12

**Authors:** Paweł Gać, Piotr Macek, Małgorzata Poręba, Olga Kornafel-Flak, Grzegorz Mazur, Rafał Poręba

**Affiliations:** 1 Centre for Diagnostic Imaging, 4th Military Hospital, Weigla 5, 50-981 Wroclaw, PL Poland; 2 Department of Hygiene, Wroclaw Medical University, Mikulicza-Radeckiego 7, 50-368 Wrocław, PL Poland; 3 Department of Internal Medicine, Occupational Diseases and Hypertension, Wroclaw Medical University, Borowska 213, 50-556 Wrocław, PL Poland; 4 Department of Pathophysiology, Wroclaw Medical University, Marcinkowskiego 1, 50-368 Wroclaw, PL Poland

**Keywords:** Coronary artery calcium score, Coronary artery disease, Epicardial adipose tissue thickness, Pericoronary adipose tissue thickness

## Abstract

**Aim:**

Determination the relationship between the epicardial adipose tissue thickness (EATT) and pericoronary adipose tissue thickness (PATT) and the risk of significant coronary artery diseases (CAD) using the coronary artery calcium score (CACS).

**Materials and methods:**

The study group consisted of 80 patients. The risk of significant CAD was estimated based on CACS. Adipose tissue thickness was measured based on multiplanar reformation (MPR), left ventricle short axis and mid-chamber level. EATT in the middle of the length of the right ventricular free wall, PATT around the left anterior descending (LAD), around the left circumflex (LCX) and around the right coronary artery in the posterior interventricular sulcus (RCA).

**Results:**

The median (IQR) values of CACS and EATT were 12.00 (97.90) and 8.65 (3.90) mm. It was found that in the subgroup CACS = 0 statistically significantly lower than in the subgroup CACS > 0 were mean values EATT and PATT RCA. Based on the regression analysis, it was demonstrated that higher CACS is associated with higher EATT, independent of older age and higher BMI. On the basis the ROC curve analysis, the highest prediction sensitivity of 98.4% was demonstrated for EATT ≥ 16.7 mm as a predictor of high risk of significant CAD and the highest specificity of 61.5% for the criterion EATT ≤ 8.7 mm as a predictor of practically no risk of significant CAD.

**Conclusion:**

There is a positive relationship between the risk of a significant CAD estimated based on the coronary artery calcium score and the epicardial adipose tissue thickness.

## Introduction

Coronary artery disease (CAD) and its complications comprise one of the main death reasons in general population [1]. Coronary heart disease, when considered separately, accounts for about 20% of all deaths, annually [2].

The fundamental diagnostic method of significant coronary artery stenosis is coronary angiography. Currently, the relevance of coronary computed tomography angiography (CCTA) as the method of diagnosing of significant coronary artery disease is growing. CCTA is a highly sensitive technique for detecting coronary artery calcium and is being used with increasing frequency for the screening of asymptomatic people to assess those at high risk for developing coronary heart disease (CHD) and cardiac events, as well as for the diagnosis of obstructive CAD in symptomatic patients [3]. The use of CCTA has the greatest potential for further determination of risk, particularly in elderly asymptomatic patients and others at intermediate risk [3]. In comparison to coronary angiography, cardiac CT has the advantage, that it is a cross-sectional technique allowing the evaluation not only of the vessel lumen but also of the vessel wall and the adjacent tissue. Thus, a more precise evaluation of coronary plaque might be obtained [4].

Coronary artery stenosis’ significance assessment can be difficult in some clinical conditions. High CACS may limit the accuracy of assessment of artery lumen [5]. Moreover, despite proper preparation, artefacts and noisy scans can occur. They are usually caused by respiratory motion, obesity, pacemakers, rapid cardiac motion (> 60 bpm) and arrhythmias [6]. Because of that, looking for additional predictors of significant coronary artery stenosis is indicated.

The calcium score index of coronary arteries (CASC) is a mathematically estimated, quantitative unit-less parameter characterizing the quantity of calcium within atherosclerotic plates in aortic walls. CASC does not directly characterize the existence of constructions in coronal arteries or their degree but enables non-invasive determination of the risk of coronary disease [7, 8].

Epicardial adipose tissue (EAT) exhibits morphological similarities with pericardial adipose tissue; however, it has different embryological origin and vascularization [9, 10]. EAT is a metabolically active organ and a major source of anti-inflammatory and proinflammatory adipokines, which have significant impact on cardiac function and morphology. Moreover, it can regulate vascular tone, by releasing various molecules [9, 10]. The relationship between the increase of EAT and cardiovascular disease is now considered a common discussion subject, being especially associated with coronary syndromes and weakening of atherosclerotic plaque [9]. According to that the thickness of the epicardial fat tissue comprises a helpful scientific parameter, which can be assessed by means of different diagnostic imaging examinations.

EAT can be visualized in echocardiography, which is a safe, easily reproducible, non-invasive and cost-effective method. For this procedure, the parasternal long and short axis views in 2D are used to achieve an accurate measurement of fat thickness in front of the free right ventricle wall [11]. In this method, difference between the epicardial and pericardial fat or between the epicardial fat and pericardial effusion can make difficulties; moreover, it does not enable the measurement of the whole volume of epicardial fat [10]. Other methods are multislice computed tomography (MSCT) and magnetic resonance (MR). Using MSCT can be measured EATT volume. In addition, information about calcification of the coronary arteries can be obtained and coronary stenosis can be assessed. The main disadvantages of MSCT are exposure to ionizing radiation and high cost [9, 10]. The gold standard for assessment of EAT volume is magnetic resonance [12]. It has better spatial resolution and enables better tissue differentiation; moreover, it lacks radiation exposure. However, it is expensive and time-consuming [9, 10].

The CACS assessment requires the use of a dedicated post-processing application. According to the authors, it is interesting to look for markers that would have a similar predictive power in the assessment of cardiovascular risk as CACS and at the same time could be easily assessed without the need to have any additional software.

The purpose of the study was to determine the relation between the epicardial adipose tissue thickness and pericoronary adipose tissue thickness measured using a 128-slice MSCT and the risk of significant CAD estimated based on the coronary artery calcium score.

## Materials and methods

The study group consisted of 80 patients of cardiological outpatient clinics referred, based on cardiovascular risk and diagnostics to date, to 128-slice CCTA. The research was conducted in the first half of 2018. The study was not available to patients after emergency CAD treatment, that is patients after PCI and/or CABG. Primary anthropometric parameters and overview of indications for CCTA in the study group are listed in Table 1.

**Table 1:** Basic clinical characteristics of the patients

	*X*	SD	Me	IQR
Age (years)	57.47	11.56	59.00	21.00
Height (m)	1.63	0.07	1.64	0.12
Body mass (kg)	73.15	10.57	75.00	16.00
BMI (kg/m^2^)	26.34	3.14	27.28	4.98
	%		*n*	
Gender				
Men	53.7		43	
Women	46.3		37	
Age				
<60 years	55.0		44	
≥60 years	45.0		36	
Body mass				
Normal	52.5		42	
Overweight/obesity	47.5		38	
Indication to CCTA				
Chronic CAD suspicion	76.2		61	
Chest pain	27.5		22	
Low intermediate CAD risk	23.7		19	
Numerous CAD risk factors	21.2		17	
Inconclusive exercise test	20.0		16	
Non-diagnostic exercise test	7.5		6	
Regional wall motion abnormalities of left ventricular	3.7		3	
Sudden cardiac death in the family history	1.2		1	

*BMI* body mass index, *CAD* coronary artery diseases, *CCTA* coronary computed tomography angiography, *IQR* interquartile range, *Me* median, *n* number, *SD* standard deviation, *X* mean

At the next stage, in the study group of patients, subgroups were isolated by criteria of age, gender and BMI. Based on the age criterion, two subgroups were isolated in the investigated group: people < 60 years (*n* = 44) and people ≥ 60 years (*n* = 36). Based on gender, another two subgroups were isolated: women (*n* = 43) and men (*n* = 37). The BMI criterion was used to isolate two more subgroups: patients with normal body mass (*n* = 42) and overweight/obese people (*n* = 38).

The research methodology included basis anthropometric measurements and CCTA. The study was approved by the Local Ethics Committee. The written informed consent was obtained from all persons taking part in the study.

The CCTA was performed using the standard protocol and dual-source 128-slice CT scanner SOMATOM Definition Dual-Source CT (Siemens Healthcare, Germany). The test protocol included the following stages: topogram, phase without applying intravenous contrast agent intended to estimate the CACS, bolus tracking, administering nitroglycerin, phase with intravenous contrast agent intended for proper assessment of the heart and coronary arteries. At the phase without intravenous contrast agent, the following technical parameters of the test: acquisition using sequential technique, craniocaudal acquisition direction from the level of trachea bifurcation to the level of diaphragm, collimation of 3.0 mm layer, exposure kilovoltage 120 units, Care Dose function (Siemens Medical Solutions, Germany), variable mAs values. Bolus tracking was performed with intravenous administration of 12 ml of contrast agent with infusion rate of 5.0 ml/s. Saturation dynamics of the proximal section of ascending aorta was assessed. Nitroglycerin was administered sublingually to achieve vasodilation of the coronary arteries, in the form of spray, in the dose of 800 g. In the phase with administration of intravenous contrast agent, the following technical parameters of the examination were applied: acquisition using spiral technique, craniocaudal acquisition direction from level 3–4 scans above the upper level of coronary arteries to the diaphragm level, collimation of layers 0.6 mm, exposure kilovoltage 120 units, variable mAs values, intravenous administration of 80–90 ml of contrast agent with infusion rate of 5.0 ml/s. Iodine, non-ionic contrast agent—iomeprol (Iomeron 400, Bracco UK Ltd, Great Britain)—was used, administered intravenously using automatic syringe through the veins of the cubital fossa.

In all the patients, based on images acquired in the CCTA, the risk of occurrence of significant CAD and the epicardial adipose tissue thickness (EATT) and the pericoronary adipose tissue thickness (PATT) were assessed.

The CACS assessment was made using an application for post-processing of computed tomography images syngo.CT CaScoring (Siemens Healthcare, Germany). The software automatically classified any lesion with size ≥ 1 mm^2^ and density ≥ 130 Hounsfield units (HU) as calcification. At the following stage, lesions classified by the application as calcified were classified as lesions within individual coronary arteries: left main (LM), left anterior descending (LAD), left circumflex (LCX) and right coronary artery (RCA). Based on the aforesaid classification, the application using the Agatston algorithm calculated the calcium score for individual coronary arteries (LM CS, LAD CS, LCX CS and RCA CS) and total CACS. The adopted final calcium score was from time to time the average of assessments made independently by two radiologists with several years’ experience in the assessment of computed tomography examinations.

The acquired CASC values were used in each patient to determine the estimated risk of significant coronary artery disease. When defining the risk of significant CAD, the following criteria were used: CACS = 0, practically no risk of significant CAD; CACS 1–10, minimal risk of significant CAD; CACS 11–100, mild risk of significant CAD; CACS 101–400, moderate risk of significant CAD; and CACS ≥ 400, high risk of significant CAD.

**Fig. 1:**
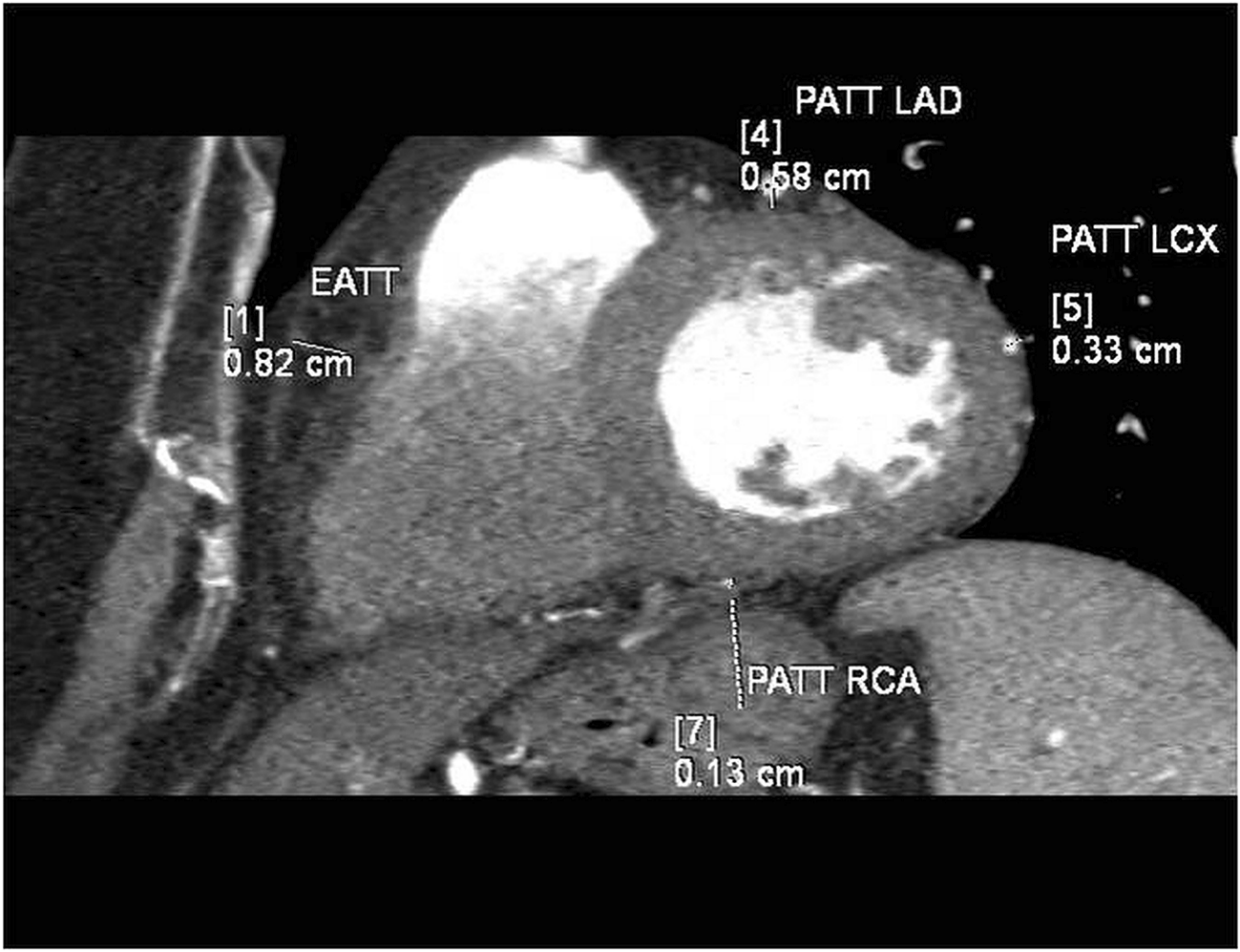
Example of EATT and PATT measurement

Assessment of the PATT and EATT was made using an application for post-processing of computed tomography images syngo.CT Cardiac Function (Siemens Healthcare, Germany). The adipose tissue thickness was measured on the basis of multiplane reconstruction (MPR) along the short axis of the left ventricle, at the level of mid-ventricular slices—the EATT at the middle length of the free right ventricle wall, the pericoronary adipose tissue thickness around the left anterior descending (PATT LAD), around left circumflex (PATT LCX) and around the right coronary artery in the posterior interventricular sulcus (PATT RCA). Every time, the final EATT and PATT values were deemed the average from independent assessments made by two radiologists with several years’ experience in the assessment of computed tomography examinations. Assessment of the EATT and PATT chronologically was an activity prior to the CACS assessment. The radiologist assessing the EATT and PATT at the time of measurement had no knowledge of CACS in the given patient. An example of EATT and PATT measurement is shown in Fig. 1.

Statistical analysis was performed using software Dell Statistica 13 (Dell Inc., USA). For quantitative variables mean, median, interquartile range and standard deviation were calculated. Lilliefors and Shapiro–Wilk tests were used to verify normal distribution of the variable’s quantitative independent variables with normal distribution were further analysed using a *t* test for independent variables. Variables with distribution other than normal were analysed using the Mann–Whitney *U* test for independent quantitative variables. Results for the quantitative variables were expressed as percentage. Independent quantitative variables were further analysed using the chi-square test for the highest reliability. To determine the relationship between the studied variables, correlation and regression analysis was performed. Due to the lack of a normal distribution of the analysed variables, non-parametric Spearman correlation coefficients were determined. Moreover, the accuracy was tested, with proposed cut-off points for the tests estimated based on the ROC (receiver operating characteristic) curves. The adopted statistical significance level was *p* < 0.05.

## Results

In the investigated group of patients, the median (IQR) value of CACS was 12.00 (97.90). Based on the CACS, the risk of significant CAD was assessed as practically non-existent (CACS = 0) for 40.0% of the subjects. The median (IQR) values of EATT, PATT LAD, PATT LCX and PATT RCA were 8.65 (3.90) mm, 7.30 (3.60) mm, 2.50 (1.50) mm and 5.85 (2.57) mm. Full assessment results for CACS, EATT and PATT in the subject group of patients are provided in Table 2.

**Table 2:** Coronary artery calcium score, epicardial adipose tissue thickness and pericoronary adipose tissue thickness in the study group

	*X*	SD	Me	IQR
CACS	154.45	357.15	12.00	97.90
LM CS	6.68	22.94	0.00	0.00
LAD CS	79.55	144.62	5.80	95.00
LCX CS	23.50	89.66	0.00	10.00
RCA CS	44.65	151.30	0.00	6.20
	%		*n*	
Significant CAD risk				
Practically non-existent	40.0		32	
Minimal	10.0		8	
Mild	25.0		20	
Moderate	13.7		11	
High	11.2		9	
	*X*	SD	Me	IQR
EATT (mm)	9.00	2.96	8.65	3.90
PATT LAD (mm)	7.71	2.80	7.30	3.60
PATT LCX (mm)	2.86	1.36	2.50	1.50
PATT RCA (mm)	6.27	2.62	5.85	2.57

*CACS* coronary artery calcium score, *CAD* coronary artery diseases, *EATT* epicardial adipose tissue thickness, *IQR* interquartile range, *LAD CS* left anterior descendens calcium score, *LCX CS* left circumflex calcium score, *LM CS* left main calcium score, *Me* median, *PATT LAD* pericoronary adipose tissue thickness around left anterior descendens, *PATT LCX* pericoronary adipose tissue thickness around left circumflex, *PATT RCA* pericoronary adipose tissue thickness around right coronary artery, *RCA CS* right coronary artery calcium score, *SD* standard deviation, *X* mean

Comparing subgroups of patients with different risk of significant CAD, it was demonstrated that the patients with practically no risk of significant CAD are characterized by significantly statistically higher EATT than the patients with minimal risk of significant CAD, patients with mild risk of significant CAD and patients with high risk of significant CAD. Moreover, the patients with high risk of significant CAD are characterized by significantly statistically higher EATT than the patients with mild risk of significant CAD and patients with moderate risk of significant CAD. Full results of the CACS, EATT and PATT assessment in the subject patients’ groups with different risk of significant CAD are presented in Table 3.

**Table 3:** Coronary artery calcium score, epicardial adipose tissue thickness and pericoronary adipose tissue thickness in the study groups distinguished based on the criterion of significant CAD risk

	Significant CAD risk					
	Practically non-existent (A, *n* = 32)	Minimal (B, *n* = 8)	Mild (C, *n* = 20)	Moderate (D, *n* = 11)	High (E, *n* = 9)	*p* < 0.05
CACS	0.00 ± 0.00/0.00 (0.00)	3.78 ± 3.30/2.40 (5.45)	40.65 ± 23.15/33.45 (22.90)	251.12 ± 102.53/254.70 (199.00)	972.36 ± 573.85/772.80 (690.40)	A, B, C vs. D A, B, C, D vs. E
LM CS	0.00 ± 0.00/0.00 (0.00)	0.15 ± 0.42/0.00 (0.00)	0.72 ± 2.38/0.00 (0.00)	12.43 ± 26.12/0.00 (13.90)	41.82 ± 50.27/30.70 (69.80)	A, B, C, D vs. E
LAD CS	0.00 ± 0.00/0.00 (0.00)	3.58 ± 3.50/2.40 (6.25)	28.88 ± 22.99/23.00 (20.70)	180.86 ± 80.99/150.80 (121.30)	413.00 ± 134.74/391.10 (131.50)	A, B, C vs. D A, B, C, D vs. E
LCX CS	0.00 ± 0.00/0.00 (0.00)	0.05 ± 0.14/0.00 (0.00)	5.02 ± 9.21/0.00 (5.20)	36.84 ± 37.66/18.10 (75.70)	150.63 ± 233.29/32.40 (71.10)	A, B, C, D vs. E
RCA CS	0.00 ± 0.00/0.00 (0.00)	0.00 ± 0.00/0.00 (0.00)	6.67 ± 13.83/0.00 (9.50)	18.91 ± 26.13/8.20 (25.90)	354.72 ± 315.35/257.50 (497.10)	A, B, C, D vs. E
EATT (mm)	7.61 ± 2.21/7.70 (2.30)	9.80 ± 4.01/10.80 (8.40)	9.49 ± 3.17/9.55 (4.30)	8.79 ± 2.12/8.95 (3.60)	11.83 ± 2.61/11.20 (2.20)	A vs. B, C, E C, D vs. E
PATT LAD (mm)	7.19 ± 3.15/6.40 (4.30)	8.74 ± 2.77/8.30 (5.80)	7.53 ± 3.00/7.50 (3.70)	8.29 ± 2.07/8.10 (3.20)	7.64 ± 2.10/7.10 (3.00)	–
PATT LCX (mm)	3.02 ± 0.83/2.90 (1.20)	2.12 ± 0.75/1.95 (1.10)	2.57 ± 1.25/2.20 (1.60)	2.77 ± 1.75/2.30 (0.50)	2.57 ± 1.10/2.40 (1.70)	–
PATT RCA (mm)	5.41 ± 1.78/5.10 (2.30)	7.27 ± 4.81/6.30 (5.30)	6.89 ± 1.94/5.55 (2.30)	6.63 ± 2.49/6.55 (4.10)	7.83 ± 3.20/7.10 (3.95)	–

Results are presented as mean ± standard deviation/median (interquartile range)

*CACS* coronary artery calcium score, *CAD* coronary artery diseases, *EATT* epicardial adipose tissue thickness, *LAD CS* left anterior descendens calcium score, *LCX CS* left circumflex calcium score, *LM CS* left main calcium score, *PATT LAD* pericoronary adipose tissue thickness around left anterior descendens, *PATT LCX* pericoronary adipose tissue thickness around left circumflex, *PATT RCA* pericoronary adipose tissue thickness around right coronary artery, *RCA CS* right coronary artery calcium score

Taking into account the predictive value of lack of calcifications in the coronary arteries (CASC = 0), the following step was to compare groups of patients with practically no risk of significant CAD (CACS = 0, *n* = 32) with a group of patients with higher-than-non-existent risk of significant CAD (CACS > 0, *n* = 48). In the entire subject group, it was found that patients with CACS = 0 are characterized by statistically significantly lower average values of EATT and PATT RCA than patients with CACS > 0. Analysing analogous results in subgroups separated based on the criterion of age, gender and BMI, it was demonstrated that significantly lower EATT in the group of patients with CACS = 0 compared with the group of patients with CACS > 0 applies to men and women, patients with normal and abnormal body mass index and patients ≥ 60 years. No such difference was demonstrated in the group of patients < 60 years. A significantly lower PATT RCA in the group of patients with CACS = 0 compared with the group of patients with CACS > 0 was observed only in patients ≥ 60 years, men and the overweight/obese. In patients < 60 years, we demonstrated a significantly lower PATT LAD in the group of patients with CACS = 0 than in the group of patients with CACS > 0. Complete results of the CACS, EATT and PATT assessments in the investigated patient groups with different risk of significant CAD are shown in Table 4.

**Table 4:** Epicardial adipose tissue thickness and pericoronary adipose tissue thickness in the study groups distinguished based on the criterion of presence of coronary artery calcifications (CACS = 0 and CACS > 0)

	Presence of coronary artery calcifications		
	CACS = 0 (practically no risk of significant CAD)	CACS > 0 (higher than no risk of significant CAD)	*p*
Whole study group			
EATT (mm)	7.61 ± 2.21/7.70 (2.30)	9.97 ± 3.11/10.20 (4.60)	< 0.05
PATT LAD (mm)	7.19 ± 3.15/6.40 (4.30)	7.93 ± 2.57/8.10 (3.10)	ns
PATT LCX (mm)	3.02 ± 0.83/2.90 (1.20)	2.55 ± 1.27/2.30 (1.40)	ns
PATT RCA (mm)	5.41 ± 1.78/5.10 (2.30)	6.65 ± 2.90/6.10 (3.90)	< 0.05
Subgroup with age < 60 years			
EATT (mm)	8.97 ± 3.10/7.70 (5.80)	9.96 ± 3.08/9.85 (4.60)	ns
PATT LAD (mm)	5.67 ± 3.67/4.40 (6.00)	7.57 ± 2.47/7.80 (2.30)	*p* < 0.05
PATT LCX (mm)	3.33 ± 0.71/3.30 (1.40)	2.72 ± 1.47/2.40 (1.70)	ns
PATT RCA (mm)	5.53 ± 0.71/5.40 (1.40)	6.42 ± 1.47/5.85 (3.60)	ns
Subgroup with age ≥ 60 years			
EATT (mm)	7.45 ± 2.11/7.80 (3.40)	10.02 ± 3.30/10.20 (3.90)	< 0.05
PATT LAD (mm)	7.36 ± 3.12/6.70 (3.12)	8.75 ± 2.71/8.30 (4.00)	ns
PATT LCX (mm)	2.96 ± 0.84/2.85 (1.20)	2.48 ± 0.66/2.45 (1.00)	ns
PATT RCA (mm)	5.40 ± 1.77/5.07 (2.60)	7.18 ± 3.66/6.30 (3.66)	< 0.05
Women			
EATT (mm)	7.59 ± 2.29/7.70 (3.40)	9.63 ± 3.09/9.70 (4.30)	< 0.05
PATT LAD (mm)	7.27 ± 3.24/6.40 (4.50)	8.13 ± 2.63/8.30 (3.30)	ns
PATT LCX (mm)	2.98 ± 0.84/2.80 (1.20)	2.44 ± 0.94/2.30 (1.40)	ns
PATT RCA (mm)	5.38 ± 1.78/5.10 (2.30)	6.01 ± 2.17/5.75 (2.45)	ns
Men			
EATT (mm)	7.85 ± 0.35/7.83 (0.50)	10.97 ± 3.08/10.90 (3.90)	< 0.05
PATT LAD (mm)	6.05 ± 1.77/6.04 (2.50)	7.34 ± 2.41/6.70 (3.20)	ns
PATT LCX (mm)	3.50 ± 0.71/3.54 (1.01)	2.91 ± 2.07/2.00 (1.50)	ns
PATT RCA (mm)	5.85 ± 2.47/5.86 (3.50)	8.51 ± 3.96/7.60 (2.90)	< 0.05
Subgroup with normal body mass (BMI < 25 kg/m^2^)			
EATT (mm)	7.27 ± 2.13/7.50 (3.50)	9.51 ± 3.33/9.90 (4.40)	< 0.05
PATT LAD (mm)	7.22 ± 3.14/6.40 (4.30)	8.65 ± 2.68/8.50 (4.00)	ns
PATT LCX (mm)	2.86 ± 0.81/2.70 (1.05)	2.24 ± 0.67/2.25 (0.90)	ns
PATT RCA (mm)	5.43 ± 1.89/5.10 (2.60)	6.06 ± 2.13/6.00 (1.30)	ns
Subgroup with overweight/obesity (BMI ≥ 25 kg/m^2^)			
EATT (mm)	8.88 ± 2.20/7.95 (3.00)	10.20 ± 3.03/10.40 (4.60)	< 0.05
PATT LAD (mm)	7.07 ± 3.52/6.55 (5.40)	7.58 ± 2.49/7.60 (2.20)	ns
PATT LCX (mm)	3.06 ± 0.59/4.00 (0.70)	2.72 ± 1.49/2.30 (1.70)	ns
PATT RCA (mm)	5.33 ± 1.44/5.15 (2.30)	6.93 ± 3.21/6.40 (3.90)	< 0.05

Results are presented as mean ± standard deviation/median (interquartile range)

*CACS* coronary artery calcium score, *CAD* coronary artery diseases, *EATT* epicardial adipose tissue thickness, *ns* non-statistically significant, *PATT LAD* pericoronary adipose tissue thickness around left anterior descendens, *PATT LCX* pericoronary adipose tissue thickness around left circumflex, *PATT RCA* pericoronary adipose tissue thickness around right coronary artery

**Fig. 2:**
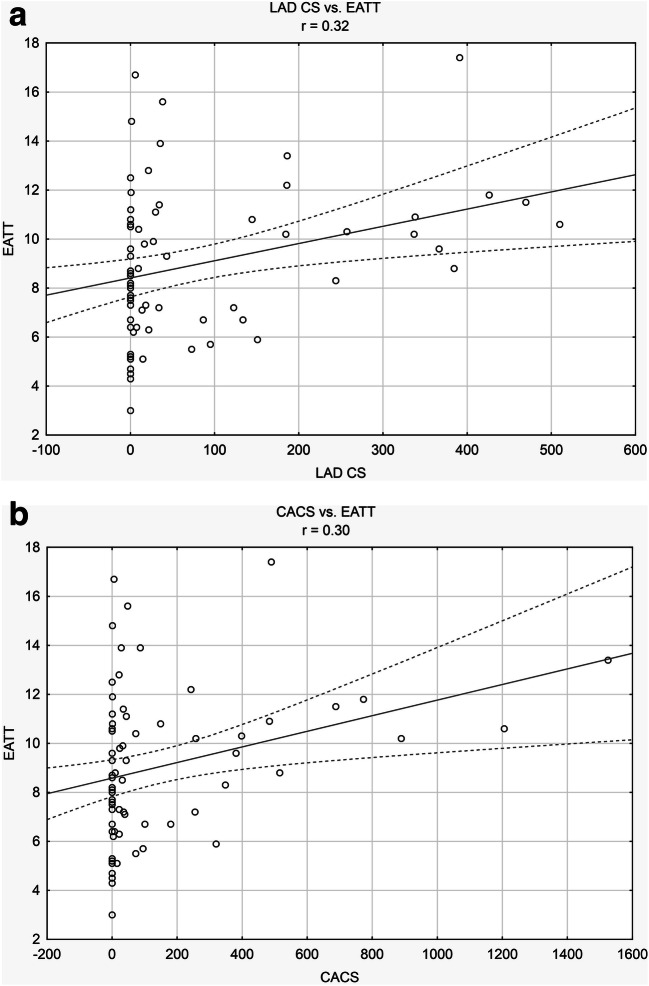
Linear relationships with the highest correlation coefficients. **a** LAD CS and EATT (*r* = 0.32, *p* < 0.05). **b** CACS and EATT (*r* = 0.30, *p* < 0.05)

The correlation analysis revealed statistically significant positive linear relationships between CACS and EATT (*r* = 0.30), LM CS as well as EATT and PATT RCA (*r* = 0.25 and *r* = 0.28, respectively), LAD CS and EATT (*r* = 0.32), LCX CS and PATT LCX (*r* = 0.26) as well as RCA CS and EATT (*r* = 0.24). Full results of the correlation analysis between the parameters of coronary arteries calcium score and the epicardial and pericoronary adipose tissue thickness are presented in Table 5. Relations with the highest correlation coefficients are presented in Fig. 2.

**Table 5:** Correlation analysis results in the study group

	EATT (mm)		PATT LAD (mm)		PATT LCX (mm)		PATT RCA (mm)	
	*r*	*p*	*r*	*p*	*r*	*p*	*r*	*p*
CACS	0.30	< 0.05	0.06	ns	0.04	ns	0.21	ns
LM CS	0.25	< 0.05	0.01	ns	− 0.04	ns	0.28	< 0.05
LAD CS	0.32	< 0.05	0.05	ns	− 0.10	ns	0.19	ns
LCX CS	0.20	ns	0.07	ns	0.26	< 0.05	0.21	ns
RCA CS	0.24	< 0.05	0.06	ns	0.01	ns	0.14	ns

*CACS* coronary artery calcium score, *EATT* epicardial adipose tissue thickness, *LAD CS* left anterior descendens calcium score, *LCX CS* left circumflex calcium score, *LM CS* left main calcium score, *ns* non-statistically significant, *PATT LAD* pericoronary adipose tissue thickness around left anterior descendens, *PATT LCX* pericoronary adipose tissue thickness around left circumflex, *PATT RCA* pericoronary adipose tissue thickness around right coronary artery, *RCA CS* right coronary artery calcium score

A multivariate regression analysis was performed to assess more accurately the relationship between CACS and EATT. The dependent variable was EATT, and the potential independent variables were CACS, age, gender (dichotomous variable: 1, male; 0, female) and body mass index. The following statistically significant regression model was obtained: EATT = − 1.155 + 0.013 CACS + 0.055 age + 0.171 BMI ± 2.414. Based on the obtained model, it was demonstrated that higher CACS is associated with higher EATT, independently of older age and higher BMI. The results of the regression analysis performed are presented in Table 6.

**Table 6:** Results of multivariate regression analysis in the study group

Model for: EATT (mm)				
	Intercept	CACS	Age (years)	BMI (kg/m^2^)
Regression coefficient	− 1.155	0.013	0.055	0.171
SEM of Rc	0.478	0.003	0.025	0.063
*p* value	0.042	0.028	0.022	0.014
*p*	< 0.01			
SEM of model	2.414			

*BMI* body mass index*, CACS* coronary artery calcium score, *EATT* epicardial adipose tissue thickness, *Rc* regression coefficient, *SEM* standard error of mean

**Fig. 3:**
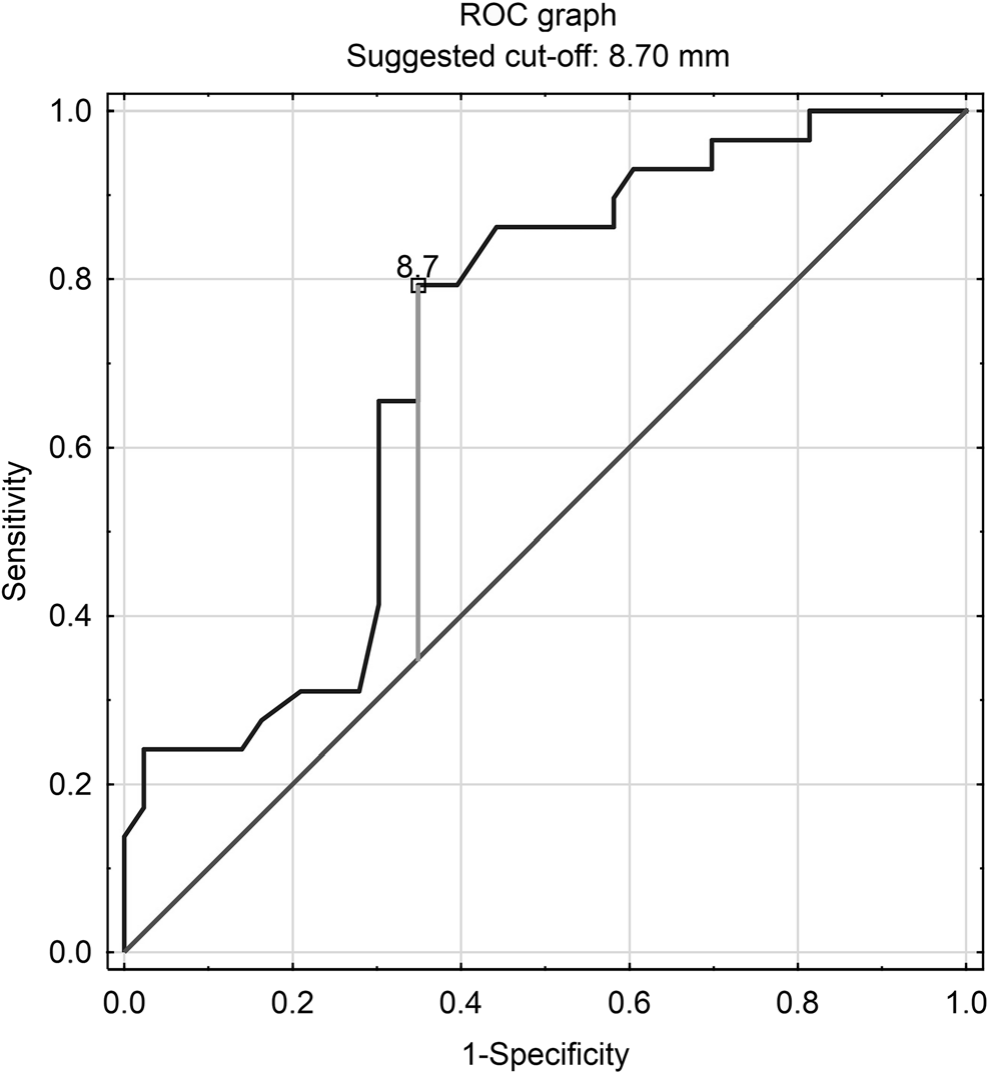
ROC prediction curve for practically non-identified risk of significant CAD using epicardial adipose tissue thickness

ROC analysis indicated the optimum EATT values, which constitute prediction factors for specific CASC values, coming to CACS = 0 to ≤ 8.7 mm, CACS > 0 to ≥ 8.8 mm, CACS > 10 to ≥ 9.8 mm, CACS > 100 to ≥ 10.2 mm and for CACS > 400 to ≥ 16.7 mm, respectively. The highest prediction sensitivity of 98.4% was demonstrated for EATT ≥ 16.7 mm as a predictor of high risk of significant CAD and the highest specificity of 61.5% for the criterion EATT ≤ 8.7 mm as a predictor of practically non-identified risk of significant CAD. In general, the highest prediction accuracy of 70.8% was obtained for the criterion EATT ≤ 8.7 mm as a predictor of practically non-identified risk of significant CAD. The ROC curve for prediction of practically non-identified risk of significant CAD among the investigated patients is shown in Fig. 3; measures of conducted connection analysis are in Table 7. In a comparative analysis, the frequency of practically non-identified risk of significant CAD was significantly statistically higher in the group of patients with EATT ≤ 8.7 mm compared with the group of patients with EATT ≥ 8.7 mm (60.5% vs. 17.6%, *p* < 0.05).

**Table 7:** The sensitivity and specificity of EATT as a predictor of CACS

	CACS = 0 (practically no risk of significant CAD)	CACS > 0 (≥ minimal risk of significant CAD)	CACS > 10 (≥ mild risk of significant CAD)	CACS > 100 (≥ moderate risk of significant CAD)	CACS > 400 (high risk of significant CAD)
EATT (mm)	≤ 8.7	≥ 8.8	≥ 9.8	≥ 10.2	≥ 17.4
Sensitivity	0.824	0.793	0.778	0.722	0.984
Specificity	0.615	0.601	0.556	0.611	0.125
Accuracy	0.708	0.706	0.667	0.694	0.689
Positive predictive values	0.651	0.605	0.636	0.848	0.900
Negative predictive values	0.793	0.824	0.714	0.423	0.500
Likelihood ratios positive	2.086	2.274	1.750	1.857	1.125
Likelihood ratios negative	0.292	0.318	0.400	0.455	0.125

*CACS* coronary artery calcium score, *CAD* coronary artery diseases, *EATT* epicardial adipose tissue thickness

## Discussion

Based on the conducted research, one should conclude that in the group of patients qualified for the examination, there is a noticeable positive relationship between the risk of a significant CAD estimated on the basis of the coronary artery calcium score and the epicardial adipose tissue thickness. The aforesaid relationship is typical of the entire population of people; otherwise, no effect on the demonstrated relationship of gender or body weight of the subject was recorded. At the same time, a significance of age was found for the analysed relationship between the coronary artery calcium score and the epicardial adipose tissue thickness. This relationship was demonstrated in the elderly population (≥ 60 years of age), whereas the analysed relationship did not occur in the younger population (< 60 years of age).

The conducted study fails to unambiguously settle the issue of relationship between the risk of a significant CAD estimated based on the coronary artery calcium score and the epicardial adipose tissue thickness. Certain positive relationships between the coronary artery calcium score and the epicardial adipose tissue thickness were demonstrated, although not in all the comparisons and analyses, as well as variable depending on the coronary artery branch at the level of which the epicardial adipose tissue thickness was measured.

Based on the results of the study, the epicardial adipose tissue thickness measured using 128-slice computed tomography can be considered a good prediction factor for the risk of significant CAD. An epicardial adipose tissue thickness ≤ 8.7 mm may be a good prediction factor for practically no risk of significant CAD, whereas EATT ≥ 16.7 mm—a good prediction factor for high risk of significant CAD.

Our study is another voice in the discussion on relationship between the coronary disease and the epicardial adipose tissue thickness measured with different methods.

Among the studies performed to date, concerning the relationship between CAD and the epicardial adipose tissue thickness, the most numerous are those where the adipose tissue thickness was measured using echocardiography. In the largest analysis available, the CAESAR study performed on a group of 2299 people, it was proven that measurement of the epicardial adipose tissue thickness is useful as an independent echocardiographic parameter, enabling detection of early and non-advanced coronary disease, estimation of its risk (just like in our study) based on the coronary artery calcium score [13]. Results which confirmed the relationship between coronary disease and epicardial adipose tissue thickness were also achieved in the studies of Cabrera-Rego et al. and Ghaderi et al. [14, 15]. In the studies of Cabrera-Rego et al. performed on a group of 300 asymptomatic Hispanic patients, it was demonstrated that the EATT measured with echocardiography rose along with the increase in CACS, whereas this relationship was only observed in the female group [14]. Ghaderi et al. demonstrated a relation between coronary disease assessed angiographically with echocardiographically measured epicardial adipose tissue thickness. A study performed on a group of 100 patients demonstrated that patients with significantly narrowed coronary arteries were characterized by significantly greater thickness of the epicardial adipose tissue, compared with the patients without significant stenosis in the coronary arteries [15]. Moreover, this study documented a relation between coronary disease risk factors and the EATT, as well as between the number of atherosclerotic coronary arteries and the EATT [15]. Yet, there are also opposing studies. After analysing data regarding 356 asymptomatic patients, Nelson at al. suggested that there is no relation between echocardiographically measured EATT and the coronary artery calcium score [16]. It should be noted that echocardiography does not enable differentiation between the epicardial and pericoronary adipose tissue, which should be deemed a limitation of this testing method.

In turn, the literature does not offer many studies seeking a relation between the epicardial or pericoronary adipose tissue and the cardiovascular risk or severity of coronary disease, using the multislice CT scans. Methodically interesting data regarding pericardial fat were provided by the study of Wang et al. [17]. These studies demonstrated that the distribution of adipose tissue around the heart is not even; this tissue was the thickest in the left atrioventricular sulcus. Based on the aforesaid study, it should be deemed necessary, in studies concerning the significance of pericardial adipose tissue, to measure the epicardial and pericoronary adipose tissue in several different anatomical locations. The result of the studies to date concerning the relation between tomographically assessed epicardial adipose tissue and coronary disease should be deemed coincident with the results of our study. In a study conducted by Goeller et al. shown, similarly to our study, that patients with CACS > 0 had significantly more epicardial adipose tissue than patients with CASC = 0 [18]. Unlike our study, the quantity of the epicardial adipose tissue was specified by semi-automatic measurement of its volume, using a software for post-processing of tomography images, instead of measuring its thickness. Also, studies by Demircelik et al. indicated a relation between the EATT and the coronary disease [19]. Yet, this study assessed the advancement of coronary disease based on an analysis of the coronary arteries’ narrowing degree in a CCTA study, while the current study focused on assessing the risk of coronary disease based on an analysis of the coronary artery calcium score. Moreover, the study by Demircelik et al., as the sole authors available, demonstrated a relation between the pericoronary adipose tissue and the advancement of CAD [19]. This relationship was not demonstrated in the current study.

In the literature to date, the matter of relationship between epicardial or pericoronary adipose tissue assessed using the magnetic resonance method and the coronary disease was only addressed occasionally. In a study by Kim et al., conducted on a group of 100 Korean patients with type 2 diabetes, without symptoms of clinically apparent coronary diseases, it was demonstrated that the EATT significantly statistically correlates with the degree of narrowing of coronary arteries. At the same time, however, no relationship between EATT and myocardial ischemic changes has been demonstrated [20].

To sum up, the meaning of the EATT thickness measurement in stratification of a significant CAD risk was, to date, demonstrated irrespective of the applied imaging method, obtaining results fundamentally coincident with the results obtained in our study. Yet, one should bear in mind the publications which negate such relationship, standing in contradiction to the results of our study. Each of the potential imaging methods is encumbered with certain limitations. In echocardiographic assessment of the epicardial adipose tissue, the problem is to determine the borders of the adipose tissue, the measurement’s dependence on the researcher’s skills and the quality of the equipment used for the examination. Moreover, it is not possible to distinguish between the epicardial and the pericoronary adipose tissue. In computer-assisted tomography, the issue is exposing the patients to ionizing radiation and iodine contrast agents. The primary flaw of magnetic resonance is the price of the test and availability of the equipment.

As a side note, it is worth to mention that usefulness of the EATT thickness measurement is also considered as a parameter of the atherosclerotic process intensification in other peripheral arteries. Recently, a relation between the EATT and the advancement of the atherosclerotic process in the carotid arteries was suggested. A relationship was proven between the EATT and the carotid intima media thickness (cIMT) [21].

The basis of pathomechanism explaining the relationship between atherosclerosis and the epicardial and pericoronary adipose tissue should be deemed the fact that the pericoronary fat has the properties of visceral adipose tissue, as well as para- and endocrine capacities. Pericoronary fat is considered a component of the visceral fat, which makes it obviously related to the metabolic syndrome and cardiovascular risk factors [22, 23]. Epicardial fat is hormonally active, it may produce and secrete at least several biologically active particles, such as adiponectin, resisting and proinflammatory cytokines, including interleukin IL-6, IL-1b and tumour necrosis factor [14, 24]. Metabolic activity of the epicardial fat may play a role in disturbing the vascular homeostasis; cause dysfunction of the vascular endothelium, acceleration of vascular wall inflammation and lesions in the intima media; and, consequently, contribute to formation of atherosclerotic plaque using the signalling mechanism “inside out” [25–27].

The results of our study regarding the relationship between CACS and EATT are generally consistent with the results of studies by other authors, as discussed above. A novel aspect of our study is the analysis of the sensitivity and specificity of EATT as a risk predictor of significant coronary artery disease. We have demonstrated that an epicardial adipose tissue thickness ≤ 8.7 mm, measured using the CCTA, may be a good prediction factor for practically no risk of significant CAD, whereas EATT ≥ 16.7 mm—a good prediction factor for high risk of significant CAD. This type of analysis has not yet been published.

Our research is not without a few limitations. The study was performed retrospectively, in a relatively small group of patients referred to a CT angiography of coronary arteries based on clinical indications. Therefore, the subject group included no asymptomatic patients, which could constitute a typical control group. For the purposes of this study, the control group was considered to consist of patients with CACS = 0 and therefore those with practically no risk of significant CAD. The study was performed on a heterogeneous population, without comparing the results with data from other centres or countries. Moreover, there are no complete clinical data available, concerning risk factors for the cardiovascular system diseases, which could be considered in more reliable models of multivariable analyses. The study does not account for actual lesions in the coronary arteries and merely assessed the risk of a significant CAD, based on the coronary artery calcium score. In addition, simple linear measurements of epicardial and peri-coronal adipose tissue thickness were used instead of the total epicardial adipose tissue volume which would be a parameter of higher representativeness due to possible asymmetric fat distribution.

### Conclusions

1. In the studied group of patients, there is a positive relationship between the risk of a significant CAD estimated based on the coronary artery calcium score and the epicardial adipose tissue thickness.
2. A positive relationship between the coronary artery calcium score and the epicardial adipose tissue was observed in the whole study group, regardless of gender and body weight, as well as in people ≥ 60 years of age. No such relationship was demonstrated in the population of people < 60 years of age.
3. An epicardial adipose tissue thickness ≤ 8.7 mm, measured using the CCTA, may be a good prediction factor for practically no risk of significant CAD, whereas EATT ≥ 16.7 mm—a good prediction factor for high risk of significant CAD.

## References

[1] GBD 2016 Causes of Death Collaborators (2017). Global, regional, and national age-sex specific mortality for 264 causes of death, 1980-2016: a systematic analysis for the Global Burden of Disease Study 2016. Lancet.

[2] Nichols M, Townsend N, Scarborough P, Rayner M (2014). Cardiovascular disease in Europe 2014: epidemiological update. Eur Heart J.

[3] O’Rourke RA, Brundage BH, Froelicher VF (2000). American College of Cardiology/American Heart Association Expert Consensus document on electron-beam computed tomography for the diagnosis and prognosis of coronary artery disease. Circulation..

[4] Aldrovandi A, Cademartiri F, Menozzi A (2008). Evaluation of coronary atherosclerosis by multislice computed tomography in patients with acute myocardial infarction and without significant coronary artery stenosis: a comparative study with quantitative coronary angiography. Circ Cardiovasc Imaging.

[5] Lee JH, Han D, Danad I (2016). Multimodality imaging in coronary artery disease: focus on computed tomography. J Cardiovasc Ultrasound.

[6] Lesser JR, Flygenring BJ, Knickelbine T (2009). Practical approaches to overcoming artifacts in coronary CT angiography. J Cardiovasc Comput Tomogr.

[7] Alluri K, Joshi PH, Henry TS (2015). Scoring of coronary artery calcium scans: history, assumptions, current limitations, and future directions. Atherosclerosis..

[8] Zeb I, Budoff M (2015). Coronary artery calcium screening: does it perform better than other cardiovascular risk stratification tools?. Int J Mol Sci.

[9] Salazar J, Luzardo E, Mejías JC (2016). Epicardial fat: physiological, pathological, and therapeutic implications. Cardiol Res Pract.

[10] Niemann M, Alkadhi H, Gotschy A (2015). Epicardial fat: imaging and implications for diseases of the cardiovascular system. Herz..

[11] Iacobellis G, Willens HJ, Barbaro G, Sharma AM (2008). Threshold values of high-risk echocardiographic epicardial fat thickness. Obesity (Silver Spring).

[12] Elming MB, Lønborg J, Rasmussen T (2013). Measurements of pericardial adipose tissue using contrast enhanced cardiac multidetector computed tomography—comparison with cardiac magnetic resonance imaging. Int J Card Imaging.

[13] Kim BJ, Kim BS, Kang JH (2015). Echocardiographic epicardial fat thickness is associated with coronary artery calcification—results from the CAESAR study. Circ J.

[14] Cabrera-Rego JO, Iacobellis G, Castillo-Herrera JA (2014). Epicardial fat thickness correlates with carotid intima-media thickness, arterial stiffness, and cardiac geometry in children and adolescents. Pediatr Cardiol.

[15] Ghaderi F, Eshraghi A, Shamloo AS, Mousavi S (2016). Assosiation of epicardial and pericardial fat thickness with coronary artery disease. Electron Physician.

[16] Nelson MR, Mookadam F, Thota V (2011). Epicardial fat: an additional measurement for subclinical atherosclerosis and cardiovascular risk stratification?. J Am Soc Echocardiogr.

[17] Wang TD, Lee WJ, Shih FY (2009). Relations of epicardial adipose tissue measured by multidetector computed tomography to components of the metabolic syndrome are region-specific and independent of anthropometric indexes and intraabdominal visceral fat. J Clin Endocrinol Metab.

[18] Goeller M, Achenbach S, Marwan M (2018). Epicardial adipose tissue density and volume are related to subclinical atherosclerosis, inflammation and major adverse cardiac events in asymptomatic subjects. J Cardiovasc Comput Tomogr.

[19] Demircelik MB, Yilmaz OC, Gurel OM (2014). Epicardial adipose tissue and pericoronary fat thickness measured with 64-multidetector computed tomography: potential predictors of the severity of coronary artery disease. Clinics (Sao Paulo).

[20] Kim HM, Kim KJ, Lee HJ (2012). Epicardial adipose tissue thickness is an indicator for coronary artery stenosis in asymptomatic type 2 diabetic patients: its assessment by cardiac magnetic resonance. Cardiovasc Diabetol.

[21] Kocaman SA, Baysan O, Çetin M (2017). An increase in epicardial adipose tissue is strongly associated with carotid-intima media thickness and atherosclerotic plaque, but LDL only with the plaque. Anatol J Cardiol.

[22] Heiss G, Sharrett AR, Barnes R (1991). Carotid atherosclerosis measured by B-mode ultrasound in populations: associations with cardiovascular risk factors in the ARIC study. Am J Epidemiol.

[23] Chambless LE, Folsom AR, Davis V (2002). Risk factors for progression of common carotid atherosclerosis: the atherosclerosis risk in communities study, 1987-1998. Am J Epidemiol.

[24] Fitzgibbons TP, Czech MP (2014). Epicardial and perivascular adipose tissues and their influence on cardiovascular disease: basic mechanisms and clinical associations. J Am Heart Assoc.

[25] Greenstein AS, Khavandi K, Withers SB (2009). Local inflammation and hypoxia abolish the protective anticontractile properties of perivascular fat in obese patients. Circulation..

[26] Cheng VY, Dey D, Tamarappoo B (2010). Pericardial fat burden on ECG-gated non contrast CT in symptomatic patients who subsequently experience adverse cardiovascular events. JACC Cardiovasc Imaging.

[27] Mahabadi AA, Massaro JM, Rosito GA (2009). Association of pericardial fat, intrathoracic fat, and visceral abdominal fat with cardiovascular disease burden: the Framingham Heart Study. Eur Heart J.

